# Cathelicidin-WA Facilitated Intestinal Fatty Acid Absorption Through Enhancing PPAR-γ Dependent Barrier Function

**DOI:** 10.3389/fimmu.2019.01674

**Published:** 2019-07-17

**Authors:** Xin Zong, Xiaoxuan Cao, Hong Wang, Xiao Xiao, Yizhen Wang, Zeqing Lu

**Affiliations:** Key Laboratory of Animal Nutrition and Feed Science in Eastern China, Ministry of Agriculture, College of Animal Sciences, Zhejiang University, Hangzhou, China

**Keywords:** cathelicidin-WA, fatty acids, intestinal barrier, PPAR-γ, lipopolysaccharides

## Abstract

The molecular mechanisms underlying the cellular uptake of long-chain fatty acids and the regulation of this process have been debated in recent decades. Here, we established an intestinal barrier dysfunction model in mice and Caco2 cell line by Lipopolysaccharide (LPS), and evaluated the fatty acid uptake capacity of the intestine. We found that LPS stimulation restricted the absorption of long chain fatty acid (LCFA), while Cathelicidin-WA (CWA) pretreatment facilitated this physiological process. At the molecular level, our results demonstrated that the stimulatory effects of CWA on intestinal lipid absorption were dependent on cluster determinant 36 and fatty acid transport protein 4, but not fatty acid–binding protein. Further, an enhanced intestinal barrier was observed *in vivo* and *in vitro* when CWA alleviated the fatty acid absorption disorder induced by LPS stimulation. Mechanistically, peroxisome proliferator-activated receptor (PPAR-γ) signaling was considered as a key pathway for CWA to enhance LCFA absorption and barrier function. Treatment with a PPAR-γ inhibitor led to impaired intestinal barrier function and suppressed LCFA uptake. Moreover, once PPAR-γ signaling was blocked, CWA pretreatment could not maintain the stability of the intestinal epithelial cell barrier or LCFA uptake after LPS stimulation. Collectively, these findings suggested that PPAR-γ may serve as a target for specific therapies aimed at alleviating fatty acid uptake disorder, and CWA showed considerable potential as a new PPAR-γ agonist to strengthen intestinal barrier function against fatty acid malabsorption.

## Introduction

Long-chain fatty acids (LCFAs, FAs with 12–18 carbons and varying degrees of unsaturation), stored as triglycerides in the body, serve several important functions in the human body (1). First, LCFAs are one of the most important sources of energy, and could provide twice as much energy as carbohydrates and proteins on a per weight basis (2). Second, dietary LCFAs are the only source of essential fatty acids that serve as substrates for lipid biosynthesis, and protein modification (1). Intestinal fatty acid absorption is a multistep process, that is associated with fatty acid transporters on the apical membrane of enterocytes, including cluster determinant 36 (CD36), plasma membrane–associated fatty acid–binding proteins (FABP), and a family of fatty acid transport proteins (FATP) 1–6 (3–5). Because of the metabolic syndrome is related to cardiovascular disease, obesity, diabetes, and cancer, many studies have focused mainly on identifying cellular, physical/chemical, and genetic determinants of intestinal fatty acid absorption in humans and laboratory animals (6).

It is generally known that before cholesterol and fatty acid molecules can interact with their corresponding transporters for uptake and absorption, they must pass through a diffusion barrier (7, 8). Intestinal mucosa is a diffusion-limiting barrier, that simultaneously promotes nutrient and water transport while serving as a protective barrier, and neither property is absolute (9). Wang et al. (7) found that epithelial mucin1 was necessary for normal intestinal uptake and absorption of cholesterol in mice, as evidenced by a 50% reduction in cholesterol absorption efficiency in mucin1 knockout mice.

Cathelicidin peptides, a family of antimicrobial peptides, not only exhibit antimicrobial activities (10), but also function as immune regulators (11). Recently, cathelicidin peptides have been found to protect intestinal epithelial barrier function in infected mice (12, 13). Our previous study showed that cathelicidin peptide (CWA) from snake attenuated EHEC-induced inflammation and microbiota disruption in the intestine of mice (14). Nevertheless, the effects of CWA on fatty acid absorption and the underlying mechanisms remain unknown.

In this study, we characterized the effect of cathelicidin peptide CWA on intestinal LCFA absorption during Lipopolysaccharide (LPS) induced intestinal barrier dysfunction in mice and Caco-2 cells. We present new evidence implicating CWA as a potent stimulator of LCFA absorption in the intestine and explore potential mechanisms through enhancing peroxisome proliferator activated receptor (PPAR) γ- dependent barrier function.

## Materials and Methods

### Peptide Synthesis

Cathelicidin-WA (CWA) was chemically synthesized by a standard solid-phase method using Automatic Peptide Synthesizer (Aapptec, Louisville, KY, USA) from C-terminus to N-terminus according to the sequence designed by our laboratory (14). The synthetic CWA was purified by semi-preparative HPLC, achieving 96% purity of the peptide and characterized by analytical HPLC (Agilent 121 Technologies, CA, USA). The peptide was dissolved in PBS and stored at −80°C until use.

### Reagents

Ultrapure LPS from *E.coli* strain O55:B5, FD4 and 4,4-difluoro-5,7-dimethyl-4-bora-3a, and 4a-diaza-s-indacene-3-hexadecanoic acid (BODIPY-C16) were purchased from Sigma-Aldrich (St. Louis. MO, USA). Olive oil and palmitic acid were purchased from Aladdin (Shanghai, China). Tyloxapol was purchased from Sigma Aldrich (St. Louis, USA). GW9662 and Rosiglitazone were obtained from Target Molecule (Shanghai, China).The rabbit antibodies for β-actin, ZO-1, CD36, FATP4, I-FABP, PPAR-γ were purchased from Proteintech (Wuhan, China). The rabbit antibodies for occludin and claudin-1 were obtained from Abcam (Cambridge, MA, USA). The secondary antibody (goat-anti-rabbit IgG) was purchased from HuaAn (Hangzhou, China).

### Animals and Treatments

Forty C57BL/6 male mice (5~6 weeks of age) were obtained from the Laboratory Animal Center of Zhejiang University. All the mice were housed in plastic cages on a layer of wood shavings with chow diet and water ad libitum under standard conditions. The animals were allowed to acclimatize to the environment for 1 week before the experiment. The animal experimental protocol was approved by the Animal Care and Use Committee of Zhejiang University.

As shown in Table 1, the mice were randomly divided into four groups of 10 each: control, LPS treatment (10 mg/kg), CWA pretreatment (5 mg/kg), and CWA+LPS (5 mg/kg CWA pretreatment followed by 10 mg/kg LPS treatment). CWA was injected intraperitoneally once daily for 6 days, whereas the control and LPS groups were intraperitoneally injected with an equal volume of sterile saline. On day 6, mice in the LPS and CWA+LPS groups were intraperitoneally injected with LPS (10 mg/kg, 200 μL per mouse) 1 h after CWA or saline treatment, and the other groups were injected with an equal volume of saline. The lipid absorption assessment was performed 6 h after LPS or saline injection, and the mice were administered 200 μL olive oil (containing 30 mg/mL palmitic acid) by gavage and then received an intraperitoneal injection of tyloxapol (15% in saline, 0.5 g/kg) 30 min later. The mice were killed and blood samples were collected by cardiac puncture 2 h after an oral fat bolus.

**Table 1:**
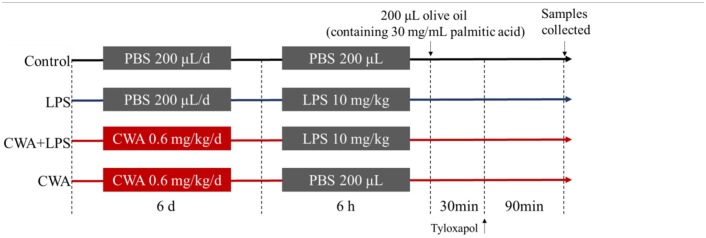
Experimental design and scheme of the animal treatments.

### Cell Culture

The human colorectal cancer cell line Caco-2 cells (a generous gift from Dr. Fengjie, College of Animal Science, Zhejiang University) were obtained from Cell Bank of the Chinese Academy of Sciences (Shanghai, China) and cultured in DMEM-F12 medium supplemented with 10% (V/V) fetal bovine serum and antibiotics (100 U/ml penicillin and 100 μg/mL streptomycin sulfate) at 37°C in humidified incubator under 5% CO_2_ in air.

### Intestinal Morphology

The proximal jejunum of the mice was fixed in 4% paraformaldehyde and embedded by paraffin, followed by slicing and staining with hematoxylin and eosin (H&E) or oil red O staining solution. Images of the slices were obtained using the Leica DM3000 Microsystem, then the villi height and crypt depth were measured by Leica Application Suite Version 3.7.0 (Leica, Wetzlar, Germany).

### Transmission Electron Microscopy

The tight junction structure was observed by TEM as previously described (10). A jejunum specimen of ~1 cm in length was excised with a sharp scalpel and fixed in 2.5% glutaraldehyde for 4 h at 4°C, followed by fixation in osmic acid and embedding in epon. Ultrathin sections were obtained using a diamond knife and stained with uranyl acetate and lead citrate before examination by TEM (JEM-1011; JEOL USA). Digital electron micrographs were acquired with a 1,024 × 1,024 pixel CCD camera system (AMT Corp., Denver, MA).

### Serum Lipids and Inflammatory Cytokines

To determine the concentration of TNF-α and IL-6 in the serum, ELISA kits (Raybiotech, Guangzhou, China) were used. The assays were carried out according to the manufacturer's instructions.

### Determination of Fatty Acid Uptake *in vitro*

Caco2 cells were seeded in BIOCOAT Collagen I 96-well plates (black wells, clear bottom) for 1 week because of the differentiation process. The fatty acid uptake was evaluated by intracellular BODIPY-labeled fatty acid as previously described (15). Briefly, the cells starved for 1 h in MEM medium (without phenol red), which was and replaced with 100 μL of a BODIPY-C16 reaction mixture prepared in D-Hanks (5 μM C1-BODIPY-C16, 5 μM fatty-free BSA and 3.9 mM trypan blue, 50 μL MEM medium). The plates were maintained in the dark and incubated for 10 min at 37°C in 5% CO_2_. Then the intracellular fluorescence was measured by a Molecular Devices SpectraMax M5 plate reader following excitation at 485 nm and emission at 528 nm. Fatty acid uptake differences were compared by the relative fluorescence.

### Measurement of Transepithelial Electrical Resistance (TEER)

Caco-2 cells were grown on 12-mm Transwell filters (Corning, NY, USA). When the TEER of cell monolayers became stable (~3 weeks later), they are ready to be studied. Then the TEER of polarized Caco-2 cells were measured using Millicell-ERS voltohmmeter (Millipore, USA) at 1, 3, 6, 12 h after LPS stimulation. Changes were calculated as a percentage of baseline TEER (0 h).

### *In vitro* Intestinal Paracellular Permeability Assay

Immediately after the last determination of TEER value, 100 μL of 4 kDa fluorescein isothiocyanate-labeled dextran (FD4, 1 mg/mL) was added to apical chamber. The transwell plates were cultured at 37°C for 30 min, and then the concentrations of FD4 in the basolateral chamber were determined at an excitation wavelength of 480 nm and an emission wavelength of 520 nm according to the standard curve, which was established from the fluorescence of FD4 at concentration of 100, 200, 500, 1,000, 2,000, 3,000, 4,000, 5,000 pmol/L.

### RNA Extraction and Quantitative Real Time PCR (q-PCR)

Total RNA was isolated using TRIzol reagent (Invitrogen, USA). The concentration and purity of the RNA were evaluated using NanoDrop2000 (Thermo Fisher Scientific, Waltham, USA). Then 2 μg RNA was subjected to reverse transcription reaction with random primers. q-PCR was performed with SYBR Green master mix (Roche, Mannheim, Germany) using a StepOnePlus Real Time PCR systems (Applied Biosystems, Foster City, USA). The gene-specific primers for the q-PCR are listed in Table 2 and the relative mRNA expression of the target gene was determined using the 2-ΔΔCt method.

**Table 2:** Primer sequences for q-PCR.

**Gene**	**Sequence (5**^****′****^** → 3**^****′****^**)**		**Genebank No**.
IL-6	Forward:	TCCTACCCCAATTTCCAATGCT	NM_031168.2
	Reverse:	TGGTCTTGGTCCTTAGCCAC	
TNF-α	Forward:	GCTCTTCTGTCTACTGAACTTCGG	NM_013693.3
	Reverse:	ATGATCTGAGTGTGAGGGTCTGG	
Occludin	Forward:	CAGGTGAATGGGTCACCGAG	NM_008756.2
	Reverse:	CAGGCTCCCAAGATAAGCGA	
ZO-1	Forward:	GGGAAGTTACGTGCGGGAG	NM_009386.2
	Reverse:	AGTGGGACAAAAGTCCGGG	
Claudin-1	Forward:	GGCCTTGGCTGTACCTTACC	NM_016674.4
	Reverse:	GGAGCACCTTATCCCCGTTT	
ApoA-IV	Forward:	GCGTGCAGGAGAAACTCAAC	NM_007468.2
	Reverse:	GCTGGTCGATTTTTGCGGAG	
CD36	Forward:	GGCAACCAACCACAAATTAGCA	NM_007643.4
	Reverse:	AAGGCTAGGAAACCATCCACC	
FATP4	Forward:	GACTTCTCCAGCCGTTTCCA	NM_011989.5
	Reverse:	AGGACAGGATGCGGCTATTG	
I-FABP	Forward:	ATGCCCACATGCTGTAGTTGA	NM_007980.3
	Reverse:	AACCTAACCGCCTCACATGC	
DGAT-1	Forward:	TTTCCGTCCAGGGTGGTAGT	NM_010046.3
	Reverse:	ATCTTGCAGACGATGGCACC	
DGAT-2	Forward:	GGCTACGTTGGCTGGTAACT	NM_026384.3
	Reverse:	TCTTCAGGGTGACTGCGTTC	
β-actin	Forward:	TGAGCTGCGTTTTACACCCT	NM_007393.5
	Reverse:	GCCTTCACCGTTCCAGTTTTT	

### Western Blot Analysis

Total protein extracts of scraped jejunal mucosa or cells were harvested by Total Protein Extraction Kit (KeyGen BioTECH, Nanjing, China). Equivalent amounts of protein were separated by SDS-PAGE and electroblotted onto PVDF membranes (Millipore, Bedford, MA, USA) followed by blocking with 5% fat-free milk. Then membranes were incubated overnight at 4°C with primary antibodies including ZO-1, occludin, claudin-1, CD36, FATP4, I-FABP, PPARγ, and β-actin. After washing with TBST, membranes were incubated with secondary antibodies for 1 h at room temperature. The protein bands were visualized with an ECL assay kit (Servicebio, Wuhan, China) and the band intensity was quantified by Image J software.

### Statistical Analysis

All statistical calculations were performed in GraphPad Prism 7 (San Diego, USA) and expressed as the mean ± SD. Data were subjected to one-way ANOVA with Duncan's multiple range test. *P* < 0.05 was considered as statistically significant.

## Results

### CWA Prevented the LCFA Absorption Disorder in LPS-Treated Mice

**Figure 1:**
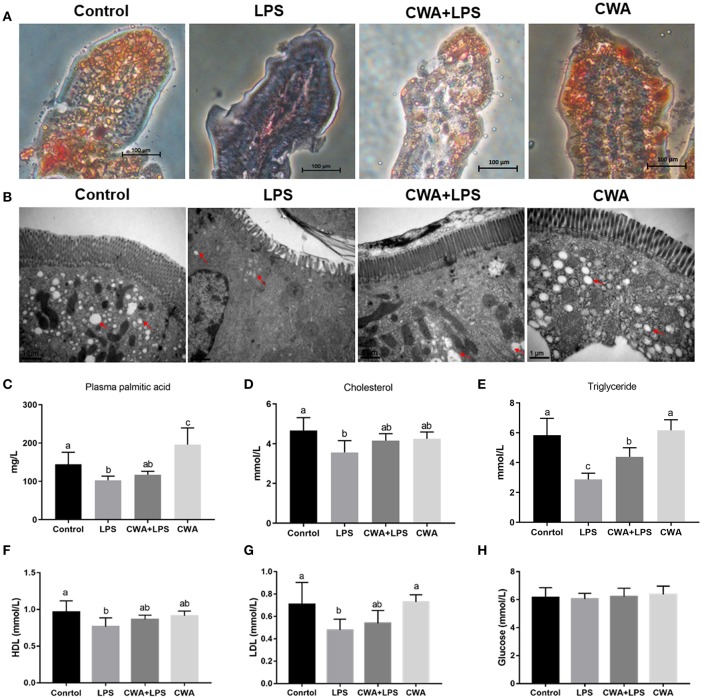
Stimulatory effects of CWA on intestinal lipid absorption. The samples were collected 6 h after LPS withdrawal and 2 h after continuous intraduodenal fat infusion in mice treated with tyloxapol. **(A)** Lipid droplets were stained with Oil red O in jejunum sections, bar = 100 μm. **(B)** Electron microscopy images showing lipid droplets in jejunum, bar = 1 μm, red arrows indicate the positions of lipid droplets. **(C)** The concentration of palmitic acid in serum was analyzed by Gas chromatography, *n* = 9, biological replicates. **(D–H)** The concentration of cholesterol **(D)**, triglyceride **(E)**, high density lipoprotein **(F)**, low density lipoprotein **(G)**, and glucose **(H)** in serum were determined by commercial kit, *n* = 9, biological replicates. The data are expressed as the mean ± SEM; bars with different small capital letters are statistically different from one another.

To directly examine whether CWA facilitates the utilization of LCFA, we measured intracellular triglyceride levels in the intestine of mice that received palmitic acid orally. As shown in the oil red O staining results (Figure 1A), some vacuolized enterocytes on the remaining villus tops in mice treated with LPS only contained a few fat droplets, while fat droplets were completely absent in the villus interstitium, which was significantly different from the control group. In contrast, in CWA-pretreated mice, most enterocytes contained no fat droplets while many small fat droplets were present in the villus interstitium. As an independent validation, a similar phenomenon was found by TEM observation (Figure 1B).

To substantiate this finding further, we found that the level of palmitic acid was not different in LPS-treated mice with or without CWA stimulation, but it increased significantly in CWA only-treated mice compared to control mice (Figure 1C). Although the levels of serum cholesterol were not changed between treatments, the level of triglycerides, the main storage form of fatty acids, in LPS-treated mice decreased markedly in comparison with control mice, and CWA pretreatment effectively alleviated the symptoms (Figures 1D,E). Moreover, we analyzed glucose and lipid metabolites in the serum. As shown in Figures 1F,G, compared with the control group, LPS stimulation decreased high-density lipoproteins (HDL) and low-density lipoproteins (LDL) levels, and CWA treatment prevented this decrease. However, it was likely that glucose metabolism was not sensitive to LPS and CWA stimulation (Figure 1H). These results indicated that LPS restricted LCFA absorption in the intestine, while CWA pretreatment restored the absorptive process during LPS stimulation.

### CWA Enhanced the Expression of Fatty Acid Absorption-Related Genes in LPS-Treated Mice

**Figure 2:**
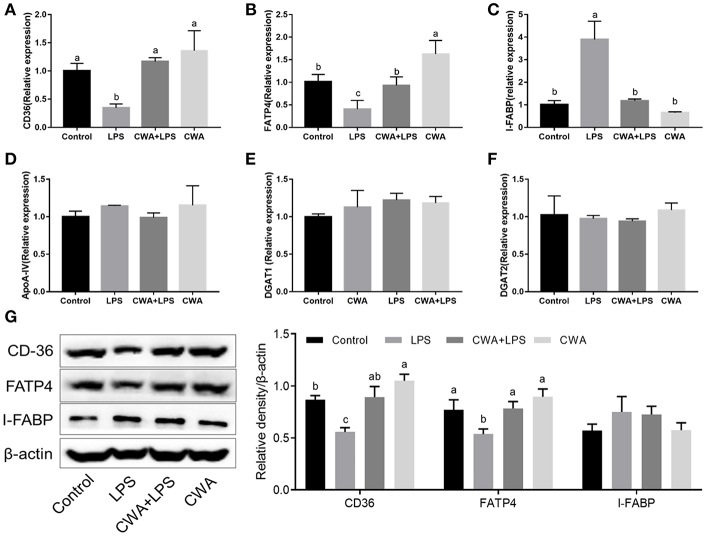
CWA enhanced the expression of fatty acid absorption related genes in LPS stimulated mice. **(A–F)** q-PCR to quantify fatty acid absorption related mRNA levels and results are presented relative to those of gapdh. **(G)** Immunoblot to determine the levels of CD36, FATP4, and I-FABP. The right panel shows the relative levels quantified by densitometry and normalized to β-actin. The data are expressed as the mean ± SEM, *n* = 9, biological replicates; bars with different small capital letters are statistically different from one another.

To investigate the genetic basis of the effect of CWA on the absorption of LCFA, the expression of specific protein transporters implicated in intestinal fatty acid absorption was examined by q-PCR. We found that the expression of CD36 and FATP4 decreased markedly after LPS stimulation, while CWA pretreatment blocked the decrease in their expression induced by LPS (Figures 2A,B). Conversely, and interestingly, compared with the control group, the expression of intestinal fatty acid binding protein (I-FABP) increased notably with LPS treatment, but no difference was noted in CWA-pretreated mice (Figure 2C). However, the expression of intestinal ApoA-IV, DGAT1, and DGAT2 upon LPS stimulation did not change among any of the treatment groups (Figures 2D–F).

Furthermore, Western blot analyses showed that exogenous CWA increased CD36 and FATP4 protein levels in mice treated with LPS, which was consistent with the qPCR results (Figure 2G). Curiously, there was no difference in the IFABP protein levels between each treatment (Figure 2G). These experiments demonstrated that the stimulatory effects of CWA on intestinal lipid absorption were dependent on the fatty acid transporters CD36 and FATP4.

### Effects of CWA on LPS-Induced Mouse Intestinal Barrier Injury

**Figure 3:**
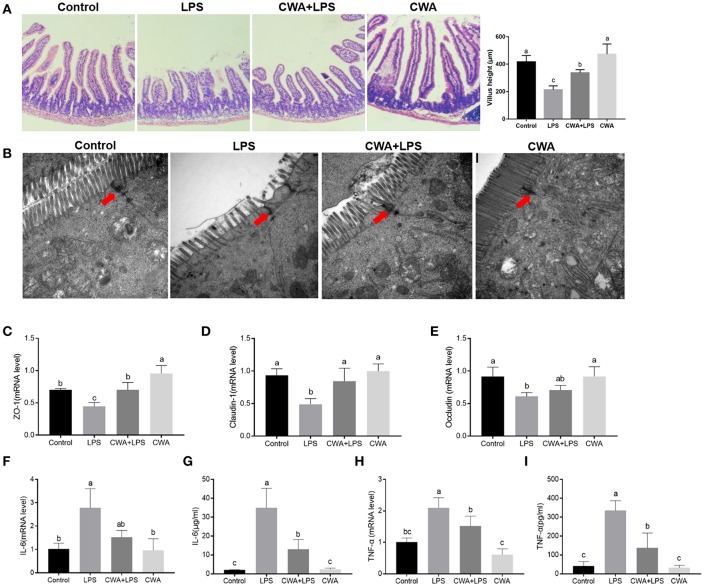
CWA prevented LPS-induced impairment of jejunum epithelium tissues. **(A)** Representative H&E-stained section from jejunum (Magnification, 100×), Villus heights of the jejunum (left; *n* = 45 for each group of mice). **(B)** Electron microscopy images showing desmosomes in intestinal epithelium (Magnification, 25000×). **(C–E)** q-PCR quantified ZO-1 **(C)**, Claudin-1 **(D)**, and Occludin **(E)** mRNA abundance in jejunum and results are presented relative to those of gapdh, *n* = 9, biological replicates. **(F–I)** q-PCR quantified mRNA abundance of IL-6 **(F)** and TNF-α **(H)** in jejunum, and Elisa determined serum concentration of IL-6 **(G)** and TNF-α **(I)**, *n* = 9, biological replicates. The data are expressed as the mean ± SEM; bars with different small capital letters are statistically different from one another.

Cathelicidin peptides improve the intestinal barrier function. We investigated whether the improved fatty acid absorption by CWA was related to its role in regulating intestinal barrier function. As shown in Figure 3A, compared with the control mice, increased villous height and decreased crypt depth were observed in the jejunum after LPS challenge, whereas CWA pretreatment attenuated the LPS-induced villous atrophy and crypt hyperplasia.

Tight junction proteins (TJs) exert a primary role in maintaining intestinal barrier function (9). To further evaluate the protective effect of CWA on the barrier function, transmission electron microscopy was used to observe TJ structure. We found that LPS destroyed the TJ structure in the jejunum, which was clearly prevented by CWA pretreatment (Figure 3B). Moreover, the expression of TJ markers, zonula occludens (ZO)−1, occludin, and Claudin-1 were detected by qPCR. All genes were downregulated in mice treated with LPS alone compared with normal animals. Administration of CWA increased the expression of all genes bringing it close to the level of the control group (Figures 3C–E). Additionally, we found that CWA attenuated the inflammatory response caused by LPS stimulation. Compared with the control group, the LPS-treated mice exhibited highly elevated expression of TNF-α and IL-6. The administration of CWA to LPS-treated mice significantly decreased these inflammatory mediators (Figures 3F–I). These results further strengthen the evidence linking CWA and intestinal barrier injury.

### CWA Prevented LCFA Malabsorption in LPS-Injured Caco-2 Cells

**Figure 4:**
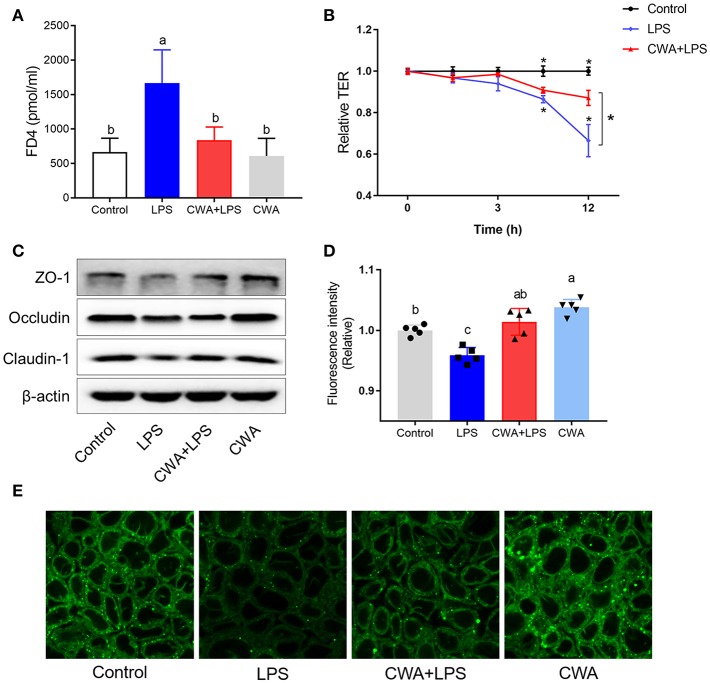
CWA (20 μg/ml) accelerated absorption of Bodipy FA in LPS injured Caco-2 cell. **(A)** Effect of CWA on cellular permeability to FD4. **(B)** Effect of CWA on TEER of a Caco-2 cell monolayer. **(C)** Western blot analysis of tight junction proteins in Caco-2 cell. **(D,E)** Effect of CWA on fatty acids uptake in Caco-2 cell. Caco-2 cells treated with medium in the presence of the Bodipy-C16 (8 μM) for 5 min. **(D)** The absorption level of Bodipy C16 was observed by confocal microscope. **(E)** General level of Bodipy FA absorption was measured by Microplate reader. The data are expressed as the mean ± SEM, *n* = 3, biological replicates; bars with different small capital letters are statistically different from one another.

To understand the mechanism by which CWA promoted LCFA absorption, a barrier-injured intestinal epithelial cell model was established with Caco-2 cells cultured in Transwell plates. Consistent with the *in vivo* findings, the results in Figure 4A show only a 20% drop in TEER values compared to the baseline values under LPS treatment conditions in the cells pretreated with CWA, whereas an ~40% decline was found in the cells treated with LPS alone (Figure 4B). The effect of CWA was further confirmed with a FD4 permeability assay (Figure 4A). At the molecular level, we verified that CWA pretreatment prevented the decreased expression of TJ markers ZO-1, occludin, and Claudin-1 induced by LPS challenge (Figure 4C). These results illustrated the protective effects of CWA on LPS-induced intestinal barrier dysfunction *in vitro*.

4,4-Difluoro-5,7-dimethyl-4-bora-3a,4a-diaza-s-indacene-3-hexadecanoic acid (Bodipy-C16) is a fatty acid analog used to study fatty acid uptake in cells (16). The incorporation of this analog into cellular lipids is similar to that of native LCFA. We used the fluorescent fatty acid analog Bodipy-C16 to measure the ability of the Caco-2 cells to take up fatty acids. Notably, upon LPS stimulation, LCFA uptake was limited, while CWA pretreatment dramatically increased the amount of fatty acid uptake when compared to the LPS-challenged group (Figure 4D). Similarly, the promotion of fatty acid uptake by CWA in cells treated with LPS was further confirmed by confocal microscopy with Bodipy-C16 (Figure 4E).

### CWA Strengthened LCFA Absorption Dependent on FATP4 and CD36

**Figure 5:**
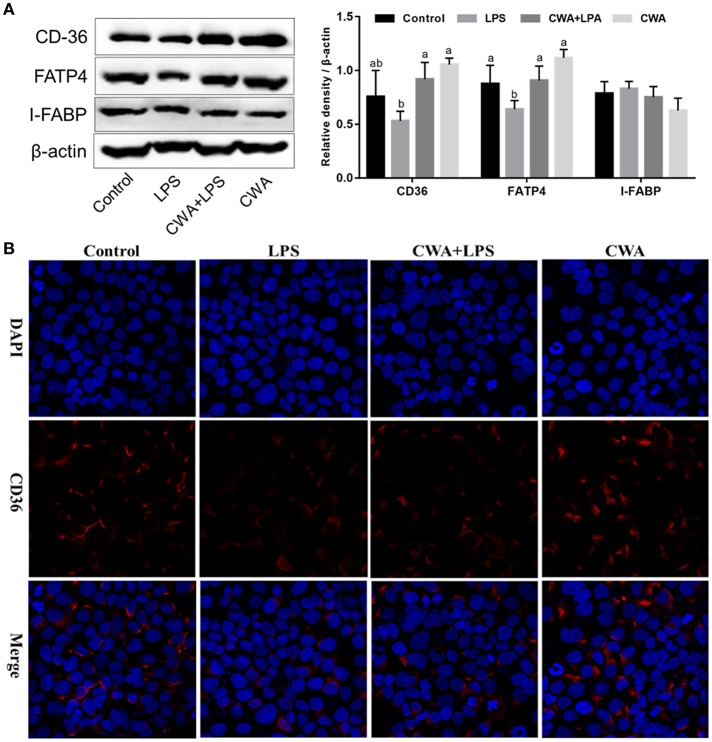
Effect of CWA on the expression and distribution of fatty acid transport proteins. **(A)** Western blot analysis of the expression of fatty acid transport protein. The right panel shows the relative levels quantified by densitometry and normalized to β-actin. **(B)** Immunofluorescence analysis of the expression and distribution of CD36. The data are expressed as the mean ± SEM, *n* = 3, biological replicates; bars with different small capital letters are statistically different from one another.

In humans and mice, CD36 is detected in epithelial cells of the small intestine along the gastrocolic and crypt-to-villus axes in a pattern paralleling that of other proteins implicated in LCFA uptake (5, 17). First, the expression of specific protein transporters implicated in intestinal fatty acid absorption was examined by Western blot analyses. Consistent with previous findings, no difference was noted in the protein levels of IFABP (Figure 5A). Moreover, we found that CWA significantly increased the expression of CD36 and FATP4, whose expression was suppressed by LPS stimulation (Figure 5A).

Immunofluorescence visualization of CD36 suggested that this protein is expressed on the membrane, poised for fatty acid uptake (Figure 5B). LPS stimulation visibly decreased the expression of CD36, which was prevented by CWA pretreatment (Figure 5B). Thus, a potential mechanism by which CWA promotes LCFA absorption is through increasing the expression of CD36 on the membrane by means of enhanced barrier function.

### CWA Facilitated LCFA Absorption and Barrier Function Through PPAR-γ Activation

**Figure 6:**
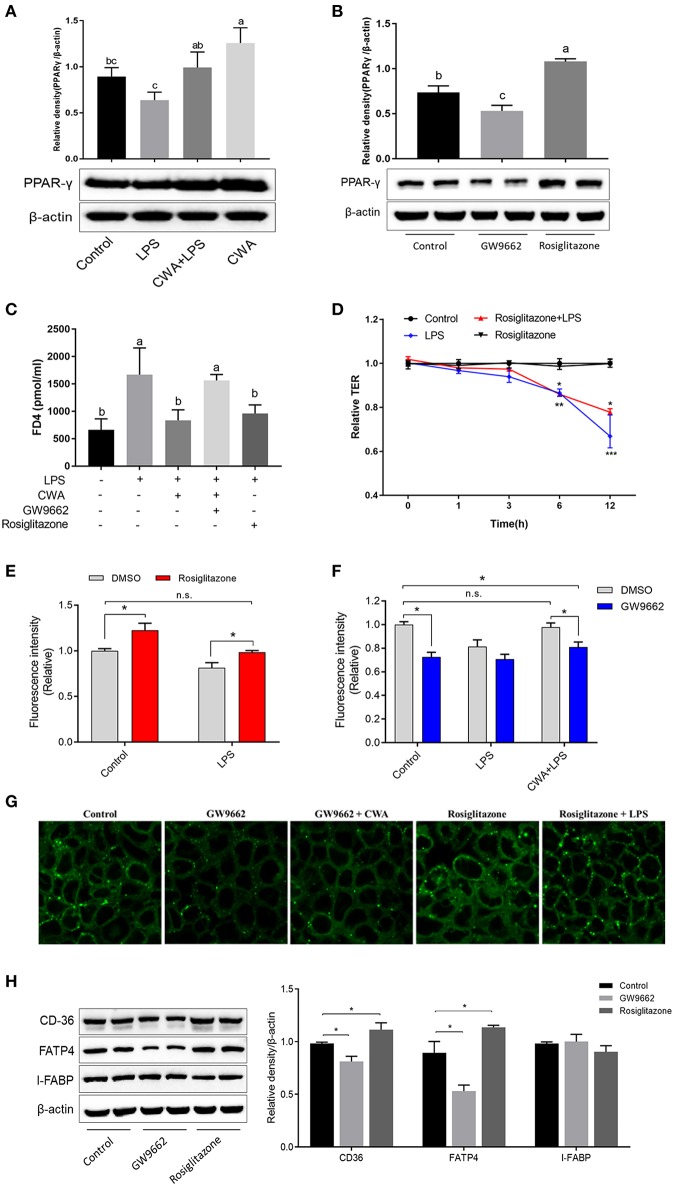
CWA facilitated intestinal fatty acid absorption and barrier function through PPAR-γ activation. **(A)** Effect of CWA on PPAR-γ activation. **(B)** Effects of GW9662 and Rosiglitazone on PPAR-γ. The top panel shows the relative levels quantified by densitometry and normalized to β-actin. **(C)** PPAR-γ is essential to the protection effects of CWA on FD4 permeation. **(D)** PPAR-γ activation ameliorates the LPS-induced TEER decrease. **(E)** PPAR-γ activation enhances intestinal fatty acid absorption. **(F)** CWA facilitated intestinal fatty acid absorption dependent on PPAR-γ activation. Fatty acid uptake was assessed by intracellular fluorescence intensity of Bodioy-C16. **(G)** CWA enhanced intestinal fatty acid absorption through activating PPAR-γ. Fatty acid uptake was assessed through intracellular fluorescence intensity of BODIPY C16 by fluorescence microscope. **(H)** Western blot analysis of the expression of fatty acid transport proteins after agonist or inhibitor of PPAR-γ treatment. The right panel shows the relative levels quantified by densitometry and normalized to β-actin. The data are expressed as the mean ± SEM, *n* = 3, biological replicates; bars with different small capital letters are statistically different from one another. ^*^P < 0.05, n.s. represented not significant.

PPAR-γ signaling is required for both intestinal barrier function and nutrient transport (18, 19). We hypothesized that CWA enhanced barrier function and that LCFA absorption was dependent on PPAR-γ. We first analyzed the expression of PPAR-γ, and as shown in Figure 6A, CWA treatment activated PPAR-γ in the presence or absence of LPS stimulation. To test the hypothesis further, an inhibitor and agonist of PPAR-γ were used. As expected, the inhibitor GW9662 inhibited and the agonist rosiglitazone activated the expression of PPAR-γ (Figure 6B). Furthermore, we found that CWA pretreatment failed to maintain the stability of the intestinal epithelial cell barrier after LPS administration when PPAR-γ signaling was inhibited (Figure 6C). Comparisons showed that the effect of CWA was similar to that of rosiglitazone (Figure 6C). These results indicated that CWA was a potential agonist of PPAR-γ. Moreover, the effect of rosiglitazone on TEER was in line with the effect of CWA, which further confirmed the conclusion that PPAR-γ is a key factor in the effect of CWA on the intestinal barrier (Figure 6D).

We further investigated the role of PPAR-γ in the process of fatty acid uptake, which is closely related to barrier function. Based on the result of fatty acid uptake ability, we found that PPAR-γ activation resulted in enhanced fatty acid uptake, regardless of LPS stimulation (Figure 6E). These findings were validated with the PPAR-γ inhibitor (Figure 6F). Next, we assessed the effects of CWA on fatty acid uptake upon LPS challenge when the expression of PPAR-γ was suppressed. As shown in Figure 6F, once the activity of PPAR-γ was inhibited, CWA was not able to maintain the absorption of fatty acids in the cells treated with LPS. In addition, the above conclusions were ascertained visually (Figure 6G). At the gene level, consistent with our hypothesis, when the activity of PPAR-γ was inhibited, the expression of FATP4 and CD36 were suppressed. Moreover, the opposite effect was observed with PPAR-γ agonists, which further verified the result (Figure 6H).

## Discussion

In our work, sufficient evidence has demonstrated that, in addition to its protective effects on the intestinal barrier, exogenous CWA may act as a PPAR-γ agonist controlling intestinal LCFA absorption. More concretely, we established LPS-induced intestinal barrier dysfunction models in mice and the Caco2 cell line and evaluated the fatty acid uptake capacity of the intestine, which still requires further investigation. Then, we discovered that CWA, a cathelicidin peptide identified in snakes, enhanced LCFA uptake, especially under the pathological state of the intestine. Finally, the molecular mechanism was elucidated, showing that CWA facilitated intestinal fatty acid absorption by enhancing PPAR-γ-dependent barrier function.

LCFAs are well-recognized as fundamental and essential nutrients for human physiology. Most LCFAs are present as esters in phospholipids and triglycerides, forms in which they perform the vital functions of membrane maintenance and energy storage. Our data suggested that CWA enhanced intestinal LCFA uptake *in vivo* and *in vitro*, which was further confirmed by the cellular triglycerides. However, CWA had no effect on the glucose balance. These results indicated that there were different absorption mechanisms between fatty acids and glucose, which was dependent on glucose transporters, such as GLUT family (20). There is debate about the overall fatty acid uptake process and whether one or more membrane-associated proteins could regulate cellular fatty acid uptake (21–23). The theory that transfer to the cytosolic leaflet of the membrane is carried out by a membrane-bound protein is the proposed pathway for the movement of LCFA into cells through the plasma membrane (24, 25). Numerous proteins have been identified for the movement of LCFA across the membrane, such as CD36, FATP, FABP, and caveolin-1 (26–28). Our data suggested that CD36 and FATP4, but not IFABP, were involved in the process of CWA regulating LCFA uptake. To our surprise, unlike the protein level, LPS stimulation significantly increased the mRNA level of IFABP. We speculated that CWA may be involved in the translation of IFABP, which still needs further study. Diacylglycerol O-acyltransferase (DGAT1) 1/2, the key enzymes in triglyceride synthesis (29), were not sensitive to CWA or LPS stimulation. The results indicated that the beneficial effects of CWA were limited to LCFA uptake.

A poorly understood feature of metabolic syndrome is that it is associated with intestinal barrier dysfunction (8, 30). The intestinal barrier is mainly composed of the mucus layer, the epithelial layer, and the underlying lamina propria. Tight junction (TJ) proteins are apical intercellular structures that connect the intestinal epithelial cells and regulate paracellular permeability (31, 32). The core TJ complex, consists of occludin, ZOs, and members of the claudin family. TJ plays a critical role in paracellular permeability by conferring selectivity to the flow of ions, small molecules and solutes between cells, which is important for the responsiveness of cells to directional stimuli and transport functions (33). Therefore, we hypothesized that CWA enhanced fatty acid absorption by strengthening barrier function. As expected, from the results of H&E, TJ structure, and the expression of a TJ marker, we found that CWA effectively attenuated LPS-induced intestinal barrier dysfunction in the jejunum. Furthermore, the results were confirmed in polarized Caco-2 cells cultured in Transwell *in vitro*. Consistent with this finding, previous studies have shown that the cathelicidin peptide cathelicidin-BF attenuates the DSS-induced disruption of mucin expression in the mouse intestine (34). Moreover, LL-37 can enhance epithelial barrier function by regulating Rac1 activation (35). This evidence indicated that the promotion of LCFA uptake by CWA was closely related to its barrier regulation function. However, the direct interaction needs to be further identified by TJ marker gene knockdown.

PPAR-γ is a member of the nuclear hormone receptor superfamily and a ligand-activated transcription factor (36, 37). It plays a critical role in the control of not only adipocyte differentiation, lipid metabolism and immunity but also the barrier functions of epithelial and endothelial cells (37–40). In the present study, in addition to CWA treatment enhancing the expression of PPAR-γ, similar effects on LCFA uptake and barrier function were also found in cells treated with CWA and the PPAR-γ agonist rosiglitazone. These results suggested that CWA may be a potential agonist of PPAR-γ. More interestingly, once PPAR-γ signaling was inhibited, CWA pretreatment could no longer maintain LCFA uptake and the barrier function after LPS stimulation. Therefore, PPAR-γ was indispensable for CWA to regulate LCFA uptake through enhancing barrier function. Taken together, our results demonstrate that exogenous CWA may thus provide a possible strategy for fatty acid absorption disorder in pathologic conditions.

## Data Availability

The datasets generated for this study are available on request to the corresponding author.

## Ethics Statement

The animal study was reviewed and approved by Animal Care and Use Committee of Zhejiang University. Written informed consent was obtained from the owners for the participation of their animals in this study.

## Author Contributions

XZ, XC, and YW designed the research. XC and HW performed experiments and collected data. XC analyzed the data. XZ wrote the manuscript. XX, YW, and ZL reviewed the manuscript.

### Conflict of Interest Statement

The authors declare that the research was conducted in the absence of any commercial or financial relationships that could be construed as a potential conflict of interest.
